# Characterization of Atherosclerotic Mice Reveals a Sex-Dependent Susceptibility to Plaque Calcification but No Major Changes in the Lymphatics in the Arterial Wall

**DOI:** 10.3390/ijms25074046

**Published:** 2024-04-05

**Authors:** Carolin Christ, Zsombor Ocskay, Gábor Kovács, Zoltán Jakus

**Affiliations:** Department of Physiology, Semmelweis University School of Medicine, 1094 Budapest, Hungary; carolin.christ@med.semmelweis-univ.hu (C.C.); ocskay.zsombor@semmelweis.hu (Z.O.); kovacs.gabor@semmelweis.hu (G.K.)

**Keywords:** lymphatics, lymphatic function, atherosclerosis, mouse models of atherosclerosis, Western diet, sex-dependent differences

## Abstract

Lymphatics participate in reverse cholesterol transport, and their presence in the arterial wall of the great vessels and prior experimental results suggest their possible role in the development of atherosclerosis. The aim of this study was to characterize the lymphatic vasculature of the arterial wall in atherosclerosis. Tissue sections and tissue-cleared aortas of wild-type mice unveiled significant differences in the density of the arterial lymphatic network throughout the arterial tree. Male and female *Ldlr^−/−^* and *ApoE^−/−^* mice on a Western diet showed sex-dependent differences in plaque formation and calcification. Female mice on a Western diet developed more calcification of atherosclerotic plaques than males. The lymphatic vessels within the aortic wall of these mice showed no major changes regarding the number of lymphatic junctions and end points or the lymphatic area. However, female mice on a Western diet showed moderate dilation of lymphatic vessels in the abdominal aorta and exhibited indications of increased peripheral lymphatic function, findings that require further studies to understand the role of lymphatics in the arterial wall during the development of atherosclerosis.

## 1. Introduction

Atherosclerosis is a chronic inflammatory disease of medium- and large-sized arteries, characterized by cholesterol accumulation and plaque formation in the arterial wall [1,2]. Smoking, diabetes mellitus, and hyperlipidemia caused by genetic factors or a Western diet and excessive food intake are the best known risk factors that can lead to the damage of the endothelium of blood vessels, inducing the influx of low-density lipoprotein (LDL) into the arterial wall [3]. LDL accumulates in the arterial wall and undergoes oxidation, a process which triggers the recruitment and adhesion of monocytes to the endothelium, where they differentiate into macrophages and engulf the oxidized LDL to become the so-called lipid laden foam cells which make up the majority of the arterial plaque [4]. As the plaque grows, foam cells die, inducing fibrosis and calcification in the necrotic core of the plaque, an indication of advanced atherosclerosis [5]. The plaque grows over decades, leading to cardiovascular complications, including aortic aneurysms, rupture of the aorta, or myocardial infarction and stroke, the most common causes of morbidity and mortality in Western societies [6,7,8]. Some of the risk factors, including a Western diet, can be eliminated by preventive measures and a healthy lifestyle. Importantly, to carry this out effectively, it is necessary to understand the pathomechanisms resulting from these risk factors.

The development of atherosclerosis can be studied in various animal models. Rabbits develop spontaneous plaques, but genetic models are rare, and their maintenance is an important limitation of their usage for these studies. Mice do not develop plaques spontaneously, but genetic mouse models have been developed to overcome this limitation. The *Ldlr^−/−^* and *ApoE^−/−^* mouse strains are two mouse models used to study atherosclerosis lacking key factors associated with lipid transport. Both develop atherosclerotic lesions throughout the arterial tree when fed a cholesterol-rich Western diet [9,10,11,12,13,14,15]. The *Ldlr^−/−^* and *ApoE^−/−^* mouse models both give different insights into atherosclerotic plaque development, like with the extremely elevated LDL levels of *Ldlr^−/−^* mice resembling more the process seen in hyperlipidemic humans [16].

Atherosclerosis has been studied extensively, but new possible factors contributing to the development of this disease continue to emerge. Some studies proposed that sex influences the progression of atherosclerosis, with female mice developing larger atherosclerotic plaques than their male counterparts [17,18,19,20]. On the contrary, in humans, women have a lower risk of developing cardiovascular disease, develop less extensive atherosclerotic plaques with less plaque ruptures, necrotic core, and calcification than men, but data are still limited regarding how biological sex-dependent differences influence the progression of atherosclerosis in mice and humans, especially focusing on its detailed pathomechanism [21,22,23].

Excess cholesterol is removed by reverse cholesterol transport (RCT), a mechanism which transports cholesterol from the periphery back to the liver for excretion via bile and stool. Impaired RCT leads to the accumulation of lipoproteins in peripheral tissues and represents a major risk factor for developing atherosclerosis [24,25,26,27]. Recent studies have shown that lymphatic vessels participate in RCT and the removal of excess cholesterol from the periphery [28,29], and these findings have led to further studies investigating the role of the lymphatic system in the development of atherosclerosis.

Lymphatics are, in fact, present in the adventitia of the arterial wall, and early studies have shown that lymphatic capillaries in the adventitia of atherosclerotic plaques are dilated in humans and mice and that their number and size increase with the progression of the disease [29,30,31,32,33,34,35]. Vuorio et al. showed that atherosclerotic lesions in mice with lymphatic deficiency progressed faster than lesions in control mice [36]. Moreover, early treatment with a lymphatic growth factor has been shown to improve lymphatic transport in 6-week-old *Ldlr^−/−^* mice and subsequently limit plaque formation [37]. In addition, other studies have also suggested the importance of lymphatics during the pathogenesis of atherosclerosis [38,39,40]. Although these results have shown that the number and size of lymphatic vessels in atherosclerosis is increased, further studies on arterial lymphatics are needed to understand their specific role in the development and progression of atherosclerosis and test whether lymphatic vasculature contributes to the sex-dependent differences in plaque formation.

In this study, we investigated atherosclerotic plaque formation in the aorta of *ApoE^−/−^* and *Ldlr^−/−^* mice. We characterized the lymphatic vasculature of the atherosclerotic arterial wall in detail. We found a sex-dependent susceptibility to atherosclerosis, with female mice developing more calcified atherosclerotic plaques than males. Detailed characterization of the lymphatic vasculature showed that the number of lymphatic junctions and end points and the lymphatic area are unaltered within the arterial wall of atherosclerotic mice. In connection, the detected moderate dilation of lymphatic vessels in the abdominal aorta and increased peripheral lymphatic function in female mice on a Western diet suggest possible sex-dependent changes in lymphatic function in atherosclerosis.

## 2. Results

### 2.1. Lymphatic Vascularization of the Arterial Wall Differs Significantly within Distinct Segments of the Aorta

**Figure 1:**
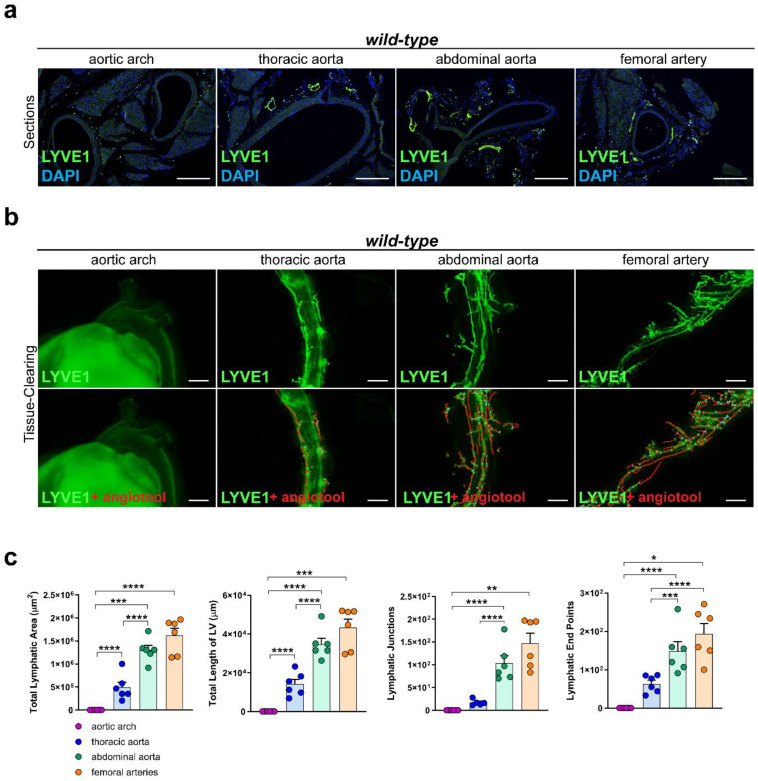
Lymphatic vasculature of the arterial wall. (**a**) Lymphatic vessels in the arterial wall of the aorta of male *C57BL/6* mice. Sections of the aortic arch, thoracic aorta, abdominal aorta, and femoral arteries were stained with the lymphatic marker anti-LYVE1. Images were acquired by upright microscopy: scale bars = 250 µm, *n* = six aortas of six mice. (**b**) Lymphatic vessels in the tissue-cleared aorta of male *C57BL/6* mice stained with anti-LYVE1. Lower images show the quantification of the lymphatic vasculature with AngioTool [42]. Images were acquired by stereo microscopy: scale bars = 1000 µm, *n* = six aortas of six mice. (**c**) Quantification of the lymphatic vasculature of the tissue-cleared aortas of male *C57BL/6* mice. The total lymphatic area, total length of LV, lymphatic junctions, and lymphatic end points were quantified using AngioTool. Data are represented as mean ± SEM and were statistically analyzed by a one-way ANOVA test; *p*-values < 0.05 were considered to be significant and indicated by asterisks: * *p* < 0.05; ** *p* < 0.01; *** *p* < 0.001; and **** *p* < 0.0001. *n* = six aortas of six mice per group. Representative images of the experiments are shown.

To investigate the lymphatic vascularization of the arterial wall, we sectioned and stained distinct segments of the aorta of *C57BL/6* wild-type mice with the lymphatic marker anti-LYVE1. We could not detect lymphatic vessels in the arterial wall of the aortic arch, while lymphatic vessels were present in the arterial wall of the other segments of the aorta, including the thoracic aorta, abdominal aorta, and femoral arteries (Figure 1a). To investigate the total coverage of lymphatic vasculature in the aorta, we tissue-cleared and stained aortas of *C57BL/6* wild-type mice with anti-LYVE1 and quantified the total lymphatic area, the length of the lymphatic vessels, the lymphatic end points, and the lymphatic junctions with AngioTool, an approach we previously described in [41]. No lymphatic structures could be detected in the arterial wall of the aortic arch, but lymphatic vessels were present in the thoracic aorta, abdominal aorta, and femoral arteries (Figure 1b). Quantifications of the images revealed significant differences regarding the total lymphatic area, the total length of the lymphatic vessels (LV), the number of lymphatic junctions, and the number of lymphatic end points within distinct segments of the aorta (Figure 1c). These results indicate that the lymphatic vascularization of the arterial wall differs significantly within distinct segments of the aorta, with few-to-no lymphatics being present in the aortic arch, more lymphatic vessels in the thoracic aorta, and most of the lymphatic vessels being present in the abdominal aorta and femoral arteries.

### 2.2. Western Diet Leads to Elevated Body Weight and Serum Lipids in Male and Female Mice

**Figure 2:**
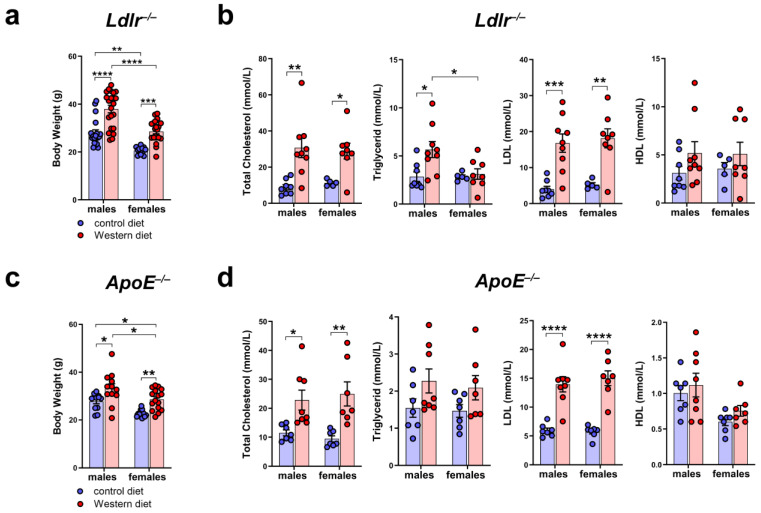
Body weight and serum cholesterol levels of *Ldlr^−/−^* and *ApoE^−/−^* mice. (**a**) Body weight of male and female *Ldlr^−/−^* mice after 20–30 weeks on a control or Western diet; *n* > 10 mice per group. (**b**) Serum cholesterol levels of male and female *Ldlr^−/−^* after 20–30 weeks on a control or Western diet; *n* = 5–9 serum samples of 5–9 mice per group. (**c**) Body weight of male and female *ApoE^−/−^* mice after 22–30 weeks on a control or Western diet; *n* > 10 mice per group. (**d**) Serum cholesterol levels of male and female *ApoE^−/−^* after 22–30 weeks on a control or Western diet. Data are represented as mean ± SEM and were statistically analyzed by a two-way ANOVA test; *p*-values < 0.05 were considered to be significant and indicated by asterisks: * *p* < 0.05; ** *p* < 0.01; *** *p* < 0.001; and **** *p* < 0.0001. *n* = 7–8 serum samples of 7–8 mice per group.

To characterize the atherosclerotic mouse models used in our study, we measured the body weight of male and female *Ldlr^−/−^* and *ApoE^−/−^* mice on a control or Western diet. The results showed that the body weight of *Ldlr^−/−^* and *ApoE^−/−^* mice increased significantly when fed a Western diet (Figure 2a,c). We could detect differences in the body weight, with male mice having a significantly higher body weight than females, both on the control and Western diets. To investigate the blood lipids of these mice, we measured the total cholesterol, triglycerides, HDL, and LDL in the serum of male and female *Ldlr^−/−^* and *ApoE^−/−^* mice on the control or Western diet. Our quantifications revealed significantly elevated total cholesterol levels in both *Ldlr^−/−^* and *ApoE^−/−^* mice on the Western diet, mainly caused by a significant increase in serum LDL levels (Figure 2b,d). Despite the much larger body weight of the males on the Western diet, we could not detect sex-dependent differences when comparing the serum lipids of males and females.

### 2.3. Female Mice Tend to Develop Larger Plaques Than Males When Fed a Western Diet

**Figure 3:**
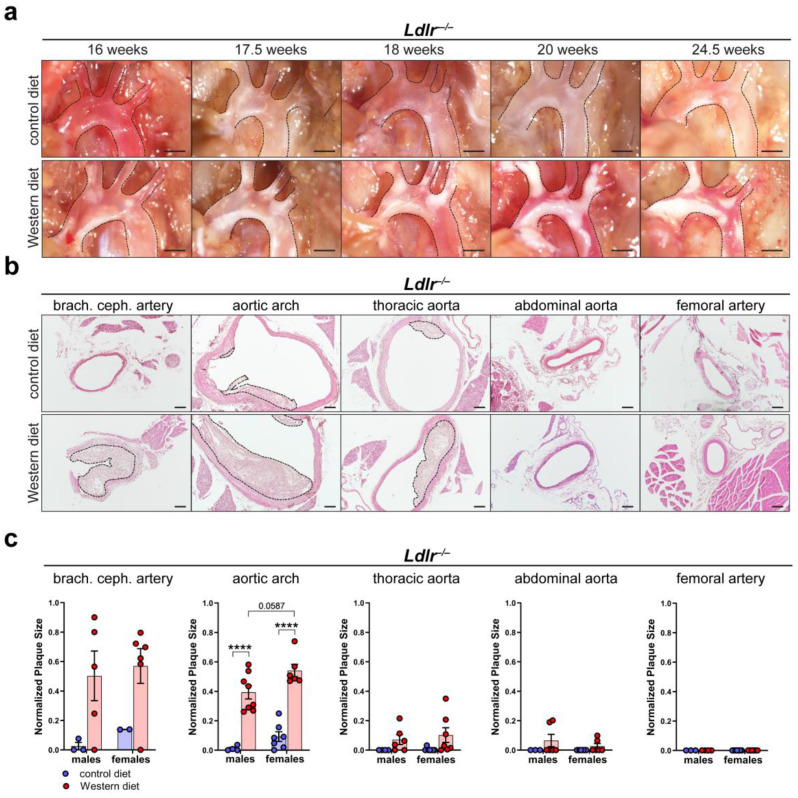
Atherosclerotic plaque development in *Ldlr^−/−^* mice. (**a**) In situ plaque development in the aortic arch of female *Ldlr^−/−^* mice on a control or Western diet at various time points. Images were acquired by stereo microscopy, scale bar = 1000 µm, *n* = 10 aortas of 10 mice. (**b**) Cross-sectional plaque area in distinct parts of the aorta of female *Ldlr^−/−^* mice after 23 weeks on a control or Western diet. Sections were stained by a H/E staining. Plaques are indicated with black dashed lines. Scale bars = 100 µm, *n* = 2–8 aortas of 2–8 mice per group. (**c**) Quantification of the cross-sectional plaque area in distinct segments of the aorta of male and female *Ldlr^−/−^* mice after 22–25 weeks on a control or Western diet. Cross-sectional plaque area was quantified manually. Data are represented as mean ± SEM and were statistically analyzed by a two-way ANOVA test (in the case of a normal distribution) or by a Kruskal–Wallis test; *p*-values < 0.05 were considered to be significant and indicated by asterisks: **** *p* < 0.0001. *n* = 2–8 aortas of 2–8 mice per group. Representative images of the experiments are shown.

To characterize atherosclerotic plaque development, we first visualized in situ plaque development in the aortic arch of *Ldlr^−/−^* mice on a control or Western diet at different time points. Lipid accumulation in the aortic arch of *Ldlr^−/−^* mice was first visible after 16 weeks and became severe after 20 weeks on the Western diet (Figure 3a). Paraffin-based histology and H/E staining of the aorta of *Ldlr^−/−^* mice on the control and Western diets were used to assess the cross-sectional plaque area in distinct segments of the aorta (Figure 3b). Manual quantifications showed that the cross-sectional plaque area of the *Ldlr^−/−^* mice on the Western diet was significantly larger than the cross-sectional plaque area of the mice on the control diet, with the largest cross-sectional plaque area being found in the brachiocephalic artery and the aortic arch (Figure 3c). No plaques were detected in the femoral arteries. Female mice on the Western diet showed a tendency to develop a larger cross-sectional plaque area in the aortic arch than the males. Further experiments indicated that *C57BL/6* control (wild-type) mice did not accumulate significant amount of lipids in the arterial wall, neither on control nor on Western diet (Supplementary Figure S2).

### 2.4. Female Mice on a Western Diet Develop Significantly Larger Plaque Calcification Than Males

**Figure 4:**
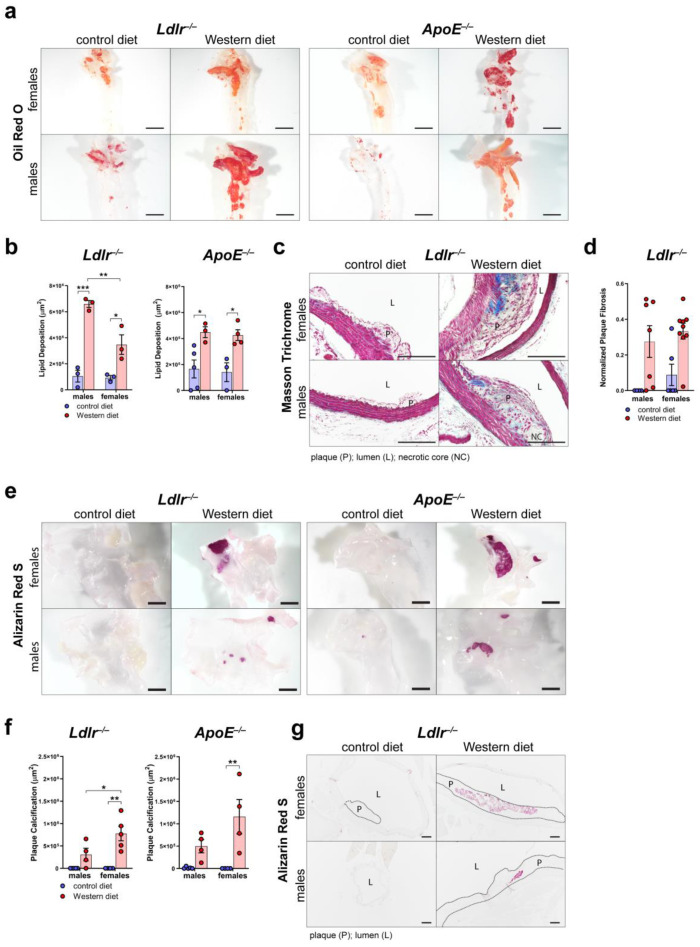
Characterization of the atherosclerotic plaques of *Ldlr^−/−^* and *ApoE^−/−^* mice. (**a**) Visualization of the lipid deposition in the arterial wall of the aortic arch of *ApoE^−/−^* and *Ldlr^−/−^* mice by Oil Red O staining after 18–30 weeks on a control or Western diet. Images were acquired by stereo microscopy: scale bar = 2500 µm, *n* = 3–5 aortas of 3–5 mice per group. (**b**) Quantification of the lipid deposition in the arterial wall of the aortic arch of *Ldlr^−/−^* and *ApoE^−/−^* mice after 18–30 weeks on a control or Western diet. Oil Red O-positive area was quantified manually. Data are represented as mean ± SEM and were analyzed by a two-way ANOVA test; *p*-values < 0.05 were considered to be significant and indicated by asterisks: * *p* < 0.05; ** *p* < 0.01; and *** *p* < 0.001. *n* = 3–5 aortas of 3–5 mice per group. (**c**) Visualization of plaque fibrosis in aortic arch sections of *Ldlr^−/−^* mice after 22–24 weeks on a control or Western diet by Masson Trichrome staining. Images were acquired by upright microscopy: scale bars = 100 µm; *n* = 4–9 aortas of 4–9 mice per group. Representative images of the experiments are shown. (**d**) Quantifications of the plaque fibrosis in male and female *Ldlr^−/−^* mice after 22–24 weeks on a control or Western diet. Masson Trichrome-positive area was quantified manually. Data are represented as mean ± SEM and were analyzed by a Kruskal–Wallis test; *p*-values < 0.05 were considered to be significant. *n* = 4–9 aortas of 4–9 mice per group. (**e**) Visualization of the plaque calcification in the aortic arch of *Ldlr^−/−^* and *ApoE^−/−^* mice after 22–26 weeks on a control or Western diet. Images were acquired by stereo microscopy: scale bar = 2500 µm, *n* = 4–5 aortas of 4–5 mice per group. Representative images of the experiments are shown. (**f**) Quantification of the calcification of the atherosclerotic plaque of *Ldlr^−/−^* and *ApoE^−/−^* mice after 22–26 weeks on a control or Western diet. Alizarin Red S-positive area was quantified manually. Data are represented as mean ± SEM and were statistically analyzed by a two-way ANOVA test; *p*-values < 0.05 were considered to be significant and were indicated by asterisks: * *p* < 0.05; and ** *p* < 0.01. *n* = 4–5 aortas of 4–5 mice per group. (**g**) Visualization of plaque calcifications in sections of the aortic arch of *Ldlr^−/−^* and *ApoE^−/−^* mice after 23–30 weeks on control and Western diets. Images were acquired by upright microscopy: scale bars = 100 µm, *n* = 4–6 aortas of 4–6 mice per group. Representative images of the experiments are shown.

To further characterize plaque formation in atherosclerosis, we first assessed the area of accumulated lipids in the arterial wall of the aortic arch of male and female *Ldlr^−/−^* and *ApoE^−/−^* mice by staining whole aortas with Oil Red O (Figure 4a). Quantifications of the Oil Red O-positive area revealed that both *Ldlr^−/−^* and *ApoE^−/−^* mice on a Western diet accumulate significantly more lipids in the arterial wall than the mice on a control diet (Figure 4b). Female *Ldlr^−/−^* mice showed a smaller lipid accumulation area in the arterial wall than the males.

To assess plaque fibrosis, we stained cross-sections of the aortic arch of *Ldlr^−/−^* mice on control and Western diets with Masson Trichrome (Figure 4c). The quantifications showed that the *Ldlr^−/−^* mice on the Western diet showed a tendency to develop larger cross-sectional fibrotic areas than the animals that had been fed the control diet (Figure 4d). More female mice on the Western diet showed large fibrotic areas of the plaques than the males, suggesting a tendency of females to develop more severe fibrosis of the atherosclerotic plaques. To investigate plaque calcification, we stained whole aortas of male and female *Ldlr^−/−^* and *ApoE^−/−^* mice with Alizarin Red S to quantify the calcified plaque area (Figure 4e). Our quantifications of the Alizarin Red S-positive area revealed that both *Ldlr^−/−^* and *ApoE^−/−^* mice on the Western diet developed severe calcification (Figure 4f). Female *Ldlr^−/−^* and *ApoE^−/−^* mice on the Western diet developed significantly larger calcifications than the females on the control diet. Female *Ldlr^−/−^* mice on the Western diet showed a significantly larger calcified area than the males, suggesting that sex could play a role in plaque calcification. Male *Ldlr^−/−^* and *ApoE^−/−^* mice showed no significant changes regarding plaque calcification when fed a Western diet. Furthermore, the largest calcified areas were found in the atherosclerotic plaques of female *Ldlr^−/−^* and *ApoE^−/−^* on the Western diet. To investigate the differences in plaque calcification further, we stained cross-sections of the aortic arch of *Ldlr^−/−^* mice on the control and Western diets with Alizarin Red S (Figure 4g). The staining of atherosclerotic lesions confirmed larger plaque calcifications in the female *Ldlr^−/−^* mice on the Western diet.

### 2.5. Number of Lymphatics and Total Lymphatic Lumen in the Arterial Wall of Atherosclerotic Mice Remain Unaltered

**Figure 5:**
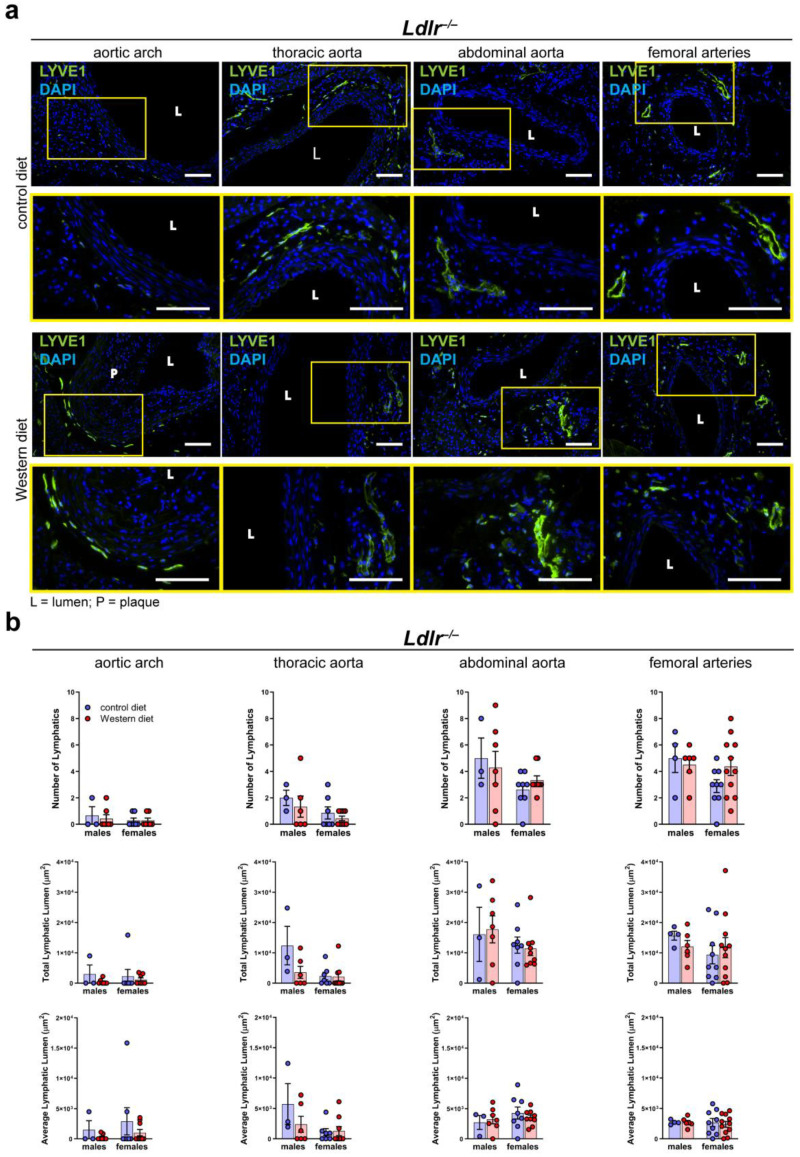
Lymphatic vessels in the arterial wall of male and female *Ldlr^−/−^* mice on a control or Western diet. (**a**) Visualization of lymphatic vessels in the adventitia of female *Ldlr^−/−^* mice after 23–30 weeks on a control or Western diet. Sections of distinct parts of the aorta were stained with the lymphatic marker anti-LYVE1. Yellow boxes on upper images mark the area shown in the lower images with higher magnification. Images were acquired by upright microscopy: scale bars = 100 µm, *n* = 2–11 aortas of 2–11 mice per group. Representative images of the experiments are shown. (**b**) Quantification of the lymphatic vessels in the adventitia of the aorta of male and female *Ldlr^−/−^* mice after 23–30 weeks on a control or Western diet. Number of lymphatics, total lymphatic lumen, and average lymphatic lumen were quantified manually. Data are represented as mean ± SEM and were statistically analyzed by a two-way ANOVA test (in the case of a normal distribution) or a Kruskal–Wallis test; *n* = 2–11 aortas of 2–11 mice per group.

To investigate the morphology of the lymphatic vasculature of the arterial wall of male and female *Ldlr^−/−^* mice on control and Western diets, we stained cross-sections of distinct segments of the aorta with the lymphatic marker anti-LYVE1, as previously described [41]. Lymphatic vessels could be detected in the thoracic, abdominal, and femoral arteries of *Ldlr^−/−^* mice on the control and Western diets (Figure 5a). As shown in the previous experiments with wild-type mice, few-to-no lymphatic vessels could be detected in the aortic wall of the aortic arch. The images showed that lymphatic vessels in the arterial wall appeared to be normal, and no major morphological changes were detected in the mice on the Western diet. Our quantifications of the lymphatic vessels in the adventitia of distinct aorta segments of the mice on the control and Western diets showed no significant differences regarding the number of lymphatics, total lymphatic lumen, or average lymphatic lumen (Figure 5b). Furthermore, no sex-dependent differences regarding the above-mentioned lymphatic parameters were detected.

### 2.6. Sex-Dependent Dilation of LVs in the Abdominal Aorta of Female Mice on a Western Diet

**Figure 6:**
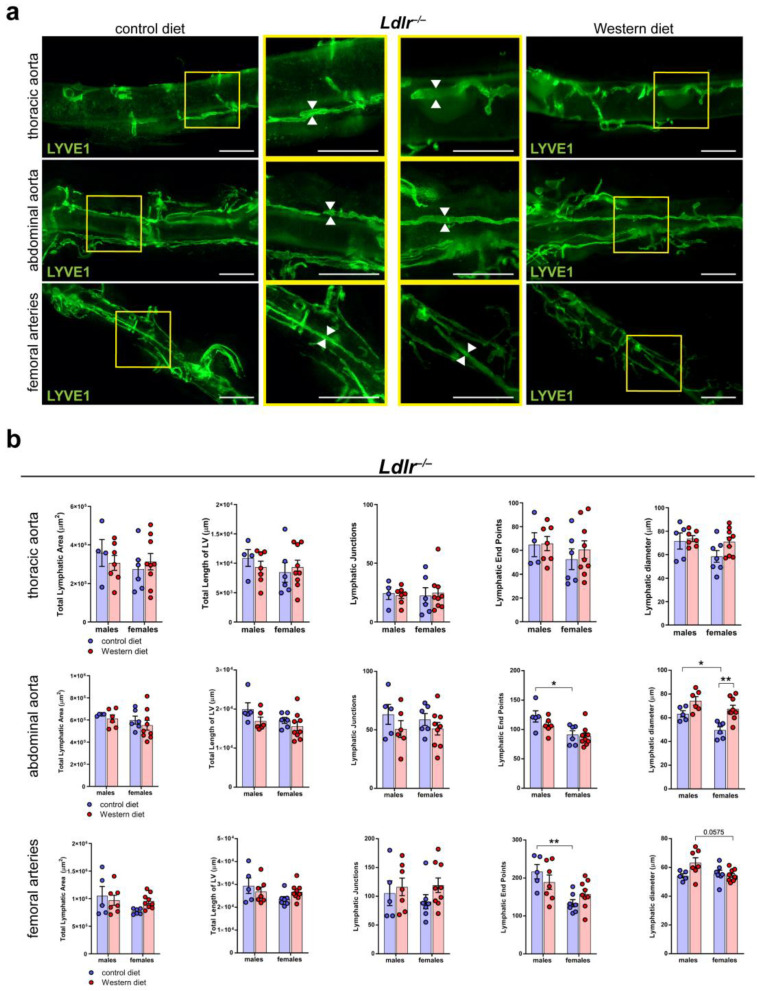
Lymphatic vasculature of the tissue-cleared arterial wall of male and female *Ldlr^−/−^* mice on a control or Western diet. (**a**) Visualization of the lymphatic vasculature of the adventitia of female *Ldlr^−/−^* mice after 21-29 weeks on a control or Western diet. Aortas were tissue-cleared and stained with the lymphatic marker anti-LYVE1. Yellow boxes on vertical images mark the area shown in the square images with higher magnification. Arrows pointing to the lymphatic vessel walls and represent the differences in the diameter of these structures. Images were acquired by stereo microscopy: scale bars = 1000 µm, *n* = 4–9 aortas of 4–9 mice per group. (**b**) Quantification of the lymphatic vasculature of the aorta of male and female *Ldlr^−/−^* mice after 21–29 weeks on a control or Western diet. Total lymphatic area, total length of lymphatic vessels, lymphatic junctions, and lymphatic end points were quantified using AngioTool, while the lymphatic diameter was manually quantified. Data are represented as mean ± SEM and were statistically analyzed by a two-way ANOVA test (in the case of a normal distribution) or a Kruskal–Wallis test. *p*-values < 0.05 were considered to be significant and indicated by asterisks: * *p* < 0.05; and ** *p* < 0.01. *n* = 4–9 aortas of 4–9 mice per group. Representative images of the experiments are shown.

To further characterize the lymphatic vasculature in atherosclerosis, we tissue-cleared aortas of *Ldlr^−/−^* mice on control and Western diets, a visualization technique we previously described in [41]. We stained the cleared aortas with the lymphatic marker anti-LYVE1 and acquired images by stereo microscopy (Figure 6a). Our quantifications of the images revealed that the Western diet led to a significant, moderate dilation of the LVs in the abdominal aorta of female *Ldlr^−/−^* mice (Figure 6b). In comparison, the Western diet led to only a slight increase in LV diameter in the abdominal aorta of male *Ldlr^−/−^* mice, suggesting a sex-dependent dilation of LVs in the aortic wall of the abdominal aorta in female mice on a Western diet.

### 2.7. No Major Changes in the LVs in Other Organs of Ldlr^−/−^ Mice on a Western Diet

**Figure 7:**
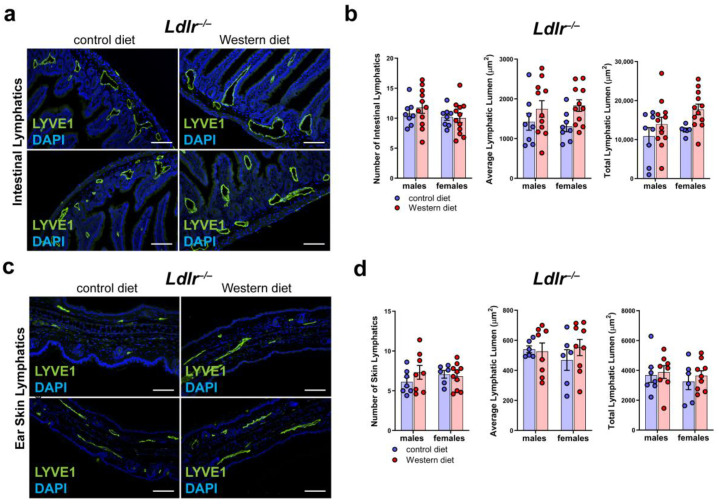
Lymphatic vasculature of the small intestine and the skin in atherosclerosis. (**a**) Visualization of the lymphatic vasculature of small intestine sections of male and female *Ldlr^−/−^* mice after 22–30 weeks on a control or Western diet. Sections were stained with the lymphatic marker anti-LYVE1. Images were acquired by upright microscopy: scale bars = 100 µm; *n* = 8–11 small intestines of 8–11 mice. Representative images of the experiments are shown. (**b**) Quantification of the organ-specific lymphatic vasculature of the small intestine of male and female *Ldlr^−/−^* mice after 22–30 weeks on a control or Western diet. Lymphatics were quantified manually. Data were statistically analyzed by a two-way ANOVA test; *p*-values < 0.05 were considered to be significant; *n* = 8–11 small intestines of 8–11 mice. (**c**) Visualization of the lymphatic vasculature of ear skin sections of male and female *Ldlr^−/−^* mice after 22–30 weeks on a control or Western diet. Sections were stained with the lymphatic marker anti-LYVE1. Images were acquired by upright microscopy: scale bars = 100 µm; *n* = 6–11 ears of 6–11 mice. Representative images of the experiments are shown. (**d**) Quantification of the organ-specific lymphatic vasculature of the skin of male and female *Ldlr^−/−^* mice after 22–30 weeks on a control or Western diet. Lymphatics were quantified manually and statistically analyzed by a two-way ANOVA test; *n* = 6–11 ears of 6–11 mice.

To see whether a Western diet has an impact on the lymphatic vasculature of other organs of *Ldlr^−/−^* mice, we sectioned and stained small intestine and skin samples with the lymphatic marker anti-LYVE1 (Figure 7a,c). Our quantifications of the lymphatic vessels in the intestine of mice on a control or Western diet revealed no changes in the number of lymphatic vessels in the small intestine and only a non-significant, slight increase in the average lymphatic lumen and total lymphatic lumen in both males and females on a Western diet (Figure 7b). The quantifications of the lymphatic vessels in the skin showed only a slight increase in the lymphatic lumen in the mice on the Western diet (Figure 7d). No sex differences in lymphatics were found in the small intestine or in the skin.

### 2.8. Increased Peripheral Lymphatic Function in Females on a Western Diet

To assess the peripheral lymphatic function in the hind limbs of *Ldlr^−/−^* mice on control and Western diets, we injected fluorescently labeled 70 kDa Rhodamine Dextran (RhD) into the hind paw of the mice and monitored the accumulation of RhD in the popliteal lymph nodes (LNs).

**Figure 8:**
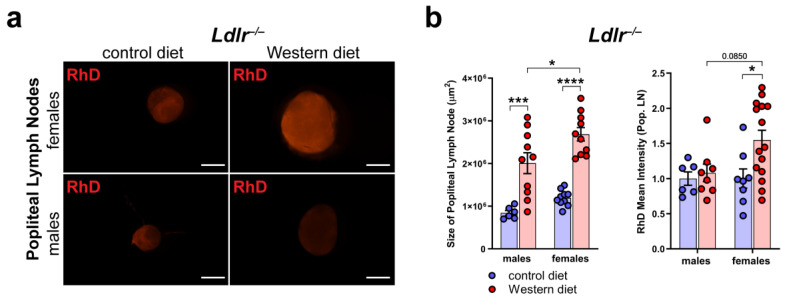
Peripheral lymphatic function in atherosclerosis. (**a**) Visualization of RhD drainage into the popliteal lymph nodes of *Ldlr^−/−^* mice after 22–30 weeks on a control or Western diet. Signal was quantified 90 min after the injection of fluorescently labeled 70 kDa RhD into the hind paws. Images were acquired by stereo microscopy: scale bars = 1000 µm; *n* = 6–12 lymph nodes of 3–6 mice per group. (**b**) Quantification of the size and RhD mean fluorescence intensity of popliteal lymph nodes of *Ldlr^−/−^* mice after 22–30 weeks on a control or Western diet. Data were quantified manually and statistically analyzed by a two-way ANOVA test. *p*-values < 0.05 were considered to be significant; * *p* < 0.05; *** *p* < 0.001; and **** *p* < 0.0001. *n* = 6–12 lymph nodes of 3–6 mice per group.

First, we dissected the popliteal LNs directly, draining the hind limb lymphatics to see if we could detect changes in the morphology and the accumulated RhD in the LNs (Figure 8a). Our quantifications showed that the size of the popliteal LNs of the *Ldlr^−/−^* mice on the Western diet was significantly increased in both male and female *Ldlr^−/−^* mice, with the popliteal LNs of the female *Ldlr^−/−^* on the Western diet being significantly larger than those of their male counterparts (Figure 8b). The measurements of the mean intensity of RhD in the popliteal LNs revealed that the female *Ldlr^−/−^* mice on the Western diet showed significantly more accumulated RhD in the popliteal LNs in comparison to the female mice on the control diet. When compared to their male counterparts, the female *Ldlr^−/−^* on the Western diet showed a tendency to accumulate more RhD in the popliteal LNs, a sign of increased peripheral lymphatic function. This result suggests a sex-dependent increase in lymphatic function in female mice on a Western diet.

## 3. Discussion

In this study, we demonstrated a sex-dependent susceptibility to atherosclerosis, with females developing more calcified plaques. Lymphatic vessels within the arterial wall are mainly unaltered in atherosclerosis but dilated in the abdominal aorta of female mice on a Western diet. These mice also show signs of increased peripheral lymphatic function, suggesting sex-dependent changes of lymphatic function in atherosclerosis.

Previous studies have linked lymphatic vessels to atherosclerosis due to their presence in the arterial wall and their newly discovered role in reverse cholesterol transport [28,29,30,31,32,33]. However, our understanding of the extent to which lymphatics contribute to the development of atherosclerosis and whether sex-dependent differences in lymphatic vasculature have an influence remains limited.

Lymphatics have been shown to be present in the adventitia of great vessels, but their function is not clear yet. Arterial lymphatics are widely believed to be beneficial in atherosclerosis by clearing cholesterol and immune cells from atherosclerotic plaques, but a new study recently showed that arterial lymphatics are one of the factors contributing to the development of transplant arteriosclerosis and that the inhibition of lymphangiogenesis would possibly prevent transplant arteriosclerosis by inhibiting the immune response [34,35,36,37,43,44,45]. Despite the fact that arterial lymphatics have been investigated in various diseases [38,39,40], a detailed characterization of the lymphatic vasculature within the arterial wall has not been carried out to date. We characterized the lymphatic vasculature of the aorta and found significant differences in the density of the arterial network throughout the arterial tree. We were unable to localize lymphatic vessels in the aortic arch, but we detected lymphatic vessels in the thoracic, abdominal, and femoral parts (Figure 1). Most of the lymphatic vessels were localized in the abdominal and femoral parts of the aorta. Differences in the lymphatic vasculature and its localization throughout the arterial tree highlight the importance of including distinct parts of the aorta when investigating the role of lymphatics in the development and progression of vascular diseases.

Furthermore, the importance of including distinct segments of the aorta in studies becomes even more evident when investigating the plaque formation that occurs in atherosclerosis. Our *Ldlr^−/−^* and *ApoE^−/−^* mouse models developed the largest atherosclerotic plaques in the brachiocephalic artery and the aortic arch when fed a Western diet (Figure 3). In contrast, we could not localize plaques in the femoral arteries and localized them only occasionally in the abdominal aorta. It is known that site-specific susceptibility to plaque formation in the aortic arch is due to hemodynamic forces, such as a low shear stress and an oscillatory or turbulent blood flow affecting the endothelium, and often appears in areas of branching or high vessel curvature [46]. The fact that the largest atherosclerotic plaques in our study were localized in the aortic arch where no lymphatic vessels could be detected might suggest a possible lymphatic contribution to the site-specific susceptibility of plaque development.

Both *Ldlr^−/−^* and *ApoE^−/−^* mice had elevated serum lipids, mainly caused by elevated LDL levels (Figure 2). These findings were consistent with previously published studies about *Ldlr^−/−^* and *ApoE^−/−^* mouse models [9,10,11,12,13,15]. The *C57BL/6* control mice did not develop atherosclerotic plaques when fed a Western diet, supporting the assumption that the loss of LDLR and the resulting hyperlipidemia are major contributors towards the development of atherosclerosis (Supplementary Figure S2) [11].

We found that the female *Ldlr^−/−^* mice showed a tendency to develop larger atherosclerotic plaques in the aortic arch than the males (Figure 3) as well as significantly larger calcification (Figure 4). In advanced atherosclerosis, smooth muscle cells or macrophages in the arterial wall undergo apoptosis and release matrix vesicles, leading to the formation of vascular calcification [5]. Calcification can usually be observed in advanced atherosclerosis and can, therefore, be used as an indicator of the severity of atherosclerosis. These findings are in line with a recent analysis of previously published atherosclerosis studies that showed that female mice show a tendency to develop larger atherosclerotic lesions than males [17,18,19]. In contrast, human epidemiological data have shown that young women below 60 years of age are less prone to develop cardiovascular disease than men but surpass them by the age of 80 [22,47].

These sex-dependent differences in the development of atherosclerosis, in both humans and animals, highlight the importance of including both sexes when studying disease development and progression to avoid biased results that could even influence the efficacy of future treatments.

Numerous studies have shown that lymphatic vessels are altered in atherosclerosis. It has been shown that the number and size of LVs are increased in atherosclerosis and that lymphatic function dependent change in the size of atherosclerotic plaques [34,37]. In this study, we could not confirm a difference in the number or size of lymphatics in the arterial wall of atherosclerotic mice and did not observe major morphological changes in the lymphatic vasculature (Figure 5). However, the tissue-cleared aortas of the female *Ldlr^−/−^* mice on the Western diet revealed dilated lymphatic vessels in the arterial wall of the abdominal aorta (Figure 6). The moderate dilation of lymphatic vessels could be a sign of altered lymphatic function in the arterial wall being involved in the removal of excess cholesterol via increased RCT. Additionally, we also found slightly dilated lymphatic vessels in the small intestine and the skin of the mice on the Western diet. These results are in line with the results of Lim et al., who proposed that lymphatic vessels are important for RCT and that moderate dilation of lymphatic vessels in the small intestine and the skin could suggest an increased RCT to remove excess cholesterol from those organs (Figure 7) [28].

Monitoring lymphatic function in the arterial wall has great limitations; therefore, we monitored and assessed peripheral lymphatic function in the hind limbs of atherosclerotic mice (Figure 8). Lymph drainage of the hind limbs of the female *Ldlr^−/−^* mice on the Western diet was significantly increased, resulting in a higher amount of accumulated RhD in the popliteal LNs. Furthermore, we found that the popliteal LNs of the female mice on the Western diet were significantly enlarged, proposing an inflammatory process in these mice. These results were in line with a study by Shin et al., who showed that the endothelial cells of female atherosclerotic mice showed a higher expression of the genes associated with inflammation and apoptosis than the males [48]. These results suggest a sex-dependent change in lymphatic function in atherosclerosis, the precise role of which is unclear and highlights the need for further investigating lymphatic function in atherosclerosis.

One of the limitations of studying lymphatics in atherosclerosis is the difficulty in monitoring lymphatic function within the great vessels in vivo or in situ. Our approach involved visualizing peripheral lymphatic function in atherosclerotic mice to investigate the impact of a Western diet on atherosclerosis. However, a better understanding of lymphatic function within great vessels would be advantageous. Due to the nature of atherosclerosis studies and experiments, spanning several months, it was impractical to incorporate diverse time points and both *ApoE^−/−^* and *Ldlr^−/−^* mice in each individual experiment. To further deepen our understanding of the role of lymphatic function in atherosclerosis development, the exploration of other atherosclerosis models, such as rabbits or larger animal models, and, ideally, human subjects would be advantageous.

In this study, we could show that lymphatic vessels are unevenly localized throughout the arterial tree and that they could contribute to site-specific susceptibility to plaque formation in atherosclerosis. We showed that lymphatic vessel morphology is mainly unaltered in atherosclerosis. Available data in the literature concerning the role of lymphatics in atherosclerosis presented diverse findings and this shows that further studies are necessary to better understand the role of lymphatics in the different aspects of atherosclerosis. The fact that female mice develop more severe cases of atherosclerosis, including more calcification in addition to enlarged LNs and increased peripheral lymphatic function, suggests the presence of a possible sex-dependent lymphatic mediated compensatory mechanism that may affect RCT and transport of immune cells.

## 4. Materials and Methods

### 4.1. Mouse Models

In this study, we used 20-weeks-old male and female *C57BL/6* control mice. Additionally, we used 23–33-weeks-old *Ldlr^−/−^* and *ApoE^−/−^* mice, well-described mouse models of atherosclerosis [9,10,11,12,13,15,49]. Both mouse models were maintained on a *C57BL/6* background. All the animals were housed under a 12/12 h light/dark cycle, with unrestricted access to food and water. All the procedures were carried out according to the Animal Experimentation Review Board of the Semmelweis University and the Government Office for Pest County, Hungary.

### 4.2. Special Diet

All the mice received either a control diet (ssniff Spezialdiäten GmbH, E15720-04, Soest, Germany) with 5% crude fat and 0% cholesterol or a Western diet (ssniff Spezialdiäten GmbH, E15721-34, Germany) with 21% crude fat and 0.21% cholesterol for 20–30 weeks. The diets started 21 days after birth, when the animals were separated from their mother. The mice had unrestricted access to food and water. The same number of mice with the same treatment period on control and Western diets was used to ensure comparability. The experimental setup shows an overview of the different experiments (Supplementary Figure S1).

### 4.3. Body Weight and Serum Cholesterol Measurements

Body weight was measured at the end of the special diet, before the mice were euthanized at the beginning of an experiment. Serum was obtained through a cardiac puncture after 24 h of starvation, centrifuged at 12,500 rpm at 4 °C, and frozen at −80 °C until measured. Total cholesterol (Beckman Coulter, OSR6116, Brea, CA, USA), triglycerides (Beckman Coulter, OSR60118), HDL (Beckman Coulter, OSR6195), and LDL (Beckman Coulter, OSR6196) were measured with an AU chemistry analyzer (Beckman Coulter, AU480) according to the protocol of the manufacturer.

### 4.4. Paraffin-Based Histology and H/E Staining of Paraffin Sections

The mice were deeply anesthetized with an intraperitoneal injection of 2.5% 2,2,2-Tribromoethanol (Sigma, T48402, St. Louis, MO, USA), followed by cardiac perfusion with 10 mL of ice cold phosphate-buffered saline (PBS)–Heparin (5000 IU/mL) and 10 mL of freshly prepared 4% paraformaldehyde (PFA). The tissue samples were collected and fixed overnight in 4% PFA at 4 °C, washed with PBS, dehydrated, and embedded in paraffin using an embedding station (Leica, EG1150H, Wetzlar, Germany). 7 μm thick sections were generated with a microtome (Thermo Fisher Scientific, HM340E, Waltham, MA, USA). The sections were deparaffinized, rehydrated, and stained according to a widely available H/E staining protocol used in previous studies [50,51,52]. Images were acquired with an upright microscope (Nikon Instruments, ECLIPSE Ni-U, Tokyo, Japan) connected to a camera (Nikon Instruments, DS-Ri2).

### 4.5. Masson Trichrome Staining of Paraffin Sections

The paraffin sections were deparaffinized, rehydrated, and stained with a Masson Trichrome Staining Kit (Sigma-Aldrich, HT15, St. Louis, MO, USA) according to the protocol of the manufacturer. Images were acquired with an upright microscope connected to a camera.

### 4.6. Immunostaining of Paraffin Sections

The paraffin sections were deparaffinized, rehydrated, and stained with the lymphatic marker goat anti-LYVE1 (R&D Systems, AF2125, Minneapolis, MN, USA) in a dilution of 1:100 and anti-goat secondary antibody conjugated to Alexa Fluor 488 (Invitrogen, A11055, Waltham, MA, USA) in a dilution of 1:250. Nuclear staining with 4′,6-Diamidino-2-phenylindole (DAPI) helped to visualize the gross morphology of the section. Images were acquired with a Nikon upright microscope connected to a camera.

### 4.7. In Situ Brightfield Images of Aortas

The mice were deeply anesthetized with an intraperitoneal injection of 2.5% 2,2,2-Tribromoethanol, followed by cardiac perfusion with 10 mL of ice cold PBS–Heparin (5000 IU/mL). Images were obtained in situ with a stereo microscope (Nikon Instruments, SMZ25, Tokyo, Japan) connected to a camera (Nikon Instruments, DS-Ri2, Tokyo, Japan) before the aortas were removed for further processing.

### 4.8. Whole-Mount Oil Red O Staining

The mice were deeply anesthetized with an intraperitoneal injection of 2.5% 2,2,2-Tribromoethanol, followed by cardiac perfusion with 10 mL of ice cold PBS–Heparin (5000 IU/mL). The aortas were dissected and fixed overnight in 4% PFA at 4 °C. The aortas were washed with PBS, immersed in 60% Isopropanol, and incubated for 15 min in an Oil Red O solution (Sigma-Aldrich, O1391, St. Louis, MO, USA) at room temperature, 80 rpm, according to the protocol of the manufacturer. The stained aortas were washed in 60% Isopropanol for 3 min and then washed with PBS. Images were acquired with a stereo microscope connected to a camera.

### 4.9. Whole-Mount Alizarin Red S Staining

Whole-mount Alizarin Red S staining was adapted from Kauffenstein et al. [53]. The mice were deeply anesthetized with an intraperitoneal injection of 2.5% 2,2,2-Tribromoethanol, followed by cardiac perfusion with 10 mL of ice cold PBS–Heparin (5000 IU/mL). The aortas were dissected and fixed overnight in 4% PFA at 4 °C. The aortas were washed with PBS and incubated in 0.003% Alizarin Red S (Sigma-Aldrich, A5533, St. Louis, MO, USA) and 1% KOH in distilled H_2_O for 48 h at room temperature, 80 rpm. The stained aortas were washed for 3 min in 1% KOH in distilled H_2_O and then washed with PBS. Images were acquired with a stereo microscope connected to a camera. The samples were stored long-term in 1:1 PBS/Glycerol (Sigma-Aldrich, G5516, St. Louis, MO, USA).

### 4.10. Tissue Clearing and Whole-Mount Immunostaining of Aortas

The tissues were cleared with our previously published approach for the visualization of organ-specific lymphatic vasculature [41]. This approach consists of a modified CUBIC protocol followed by whole-mount immunostaining with the lymphatic marker anti-LYVE1. Images were acquired by stereo microscopy and quantified with AngioTool (v0.5) [42].

### 4.11. Microscopic Imaging and Processing

Images were acquired by upright microscopy or by stereo microscopy, both connected to a camera. Images were processed and analyzed using the NIS-Elements imaging software (Nikon Instruments, version BR 4.60.00).

### 4.12. Quantification of Cross-Sectional Atherosclerotic Plaque Area

Plaque sizes were quantified manually using H/E images or immune-stained paraffin sections (4× magnification) by Fiji (ImageJ, version 1.52p) [54]. We measured the lumen of the vessel and the size of the plaque and calculated the percentage of vessel lumen blocked by the atherosclerotic plaque (normalized plaque size).

### 4.13. Manual Quantification of Lymphatic Structures in Paraffin Sections

Manual quantifications were performed using NIS-Elements. For the quantification of the sections, the lymphatic number, total lymphatic lumen, and average lymphatic lumen of one section (10× magnification) were quantified for each mouse. For the quantification of the lymphatic diameter of tissue-cleared aortas, 30 lymphatic vessels (1.5× magnification) were measured per mouse.

### 4.14. Quantifications of Whole-Mount Lymphatic Structures with AngioTool

Total lymphatic area, total length of lymphatic vessels, lymphatic junctions, and lymphatic end points were quantified with AngioTool (v0.5), a free computational tool which has been developed for the quantitative analysis of vascular networks [42]. After uploading the images to AngioTool, we adjusted parameters such as the vessel diameter and intensity and removed small articles from the calculation. All the images were quantified using the same parameters.

### 4.15. Data Representation and Statistical Analysis

Representative images of the experiments are shown. Data were processed and statistically analyzed using GraphPad Prism (version 7.03) and Excel (Microsoft, version 2018). All the datasets were analyzed to identify outliers using the robust regression and outlier removal (ROUT) method, with a Q = 5%. The data points that were identified as outliers were removed from the dataset. All the datasets were then tested for normal distribution using the Shapiro–Wilk test, with an alpha = 0.01. The datasets showing normal distribution were compared with parametric tests including a one-way or two-way analysis of variance (ANOVA), followed by Tukey’s post hoc test, while the datasets not following a normal distribution were compared with non-parametric tests, including the Kruskal–Wallis H-test, followed by Dunn’s post hoc test. An alpha of <0.05 was considered to be significant.

## Data Availability

The datasets for this study are available from the corresponding author upon reasonable request.

## Supplementary Materials

The following supporting information can be downloaded at https://www.mdpi.com/article/10.3390/ijms25074046/s1.
