# Supplementary material for: Characterization of Atherosclerotic Mice Reveals a Sex-Dependent Susceptibility to Plaque Calcification but No Major Changes in the Lymphatics in the Arterial Wall
Source: Int J Mol Sci. 2024 Apr 5;25(7):4046. doi: 10.3390/ijms25074046 (PMC11012298; doi:10.3390/ijms25074046)
Supplement: Supplementary file 1 [file ijms-25-04046-s001.zip › ijms-2887225-supplementary.pdf]

Characterization of Atherosclerotic Mice Reveals a Sex-Dependent Susceptibility to Plaque Calcification but No Major Changes in the Lymphatics in the Arterial Wall

Carolin Christ[1], Zsombor Ocskay[1], Gábor Kovács[1] and Zoltán Jakus[1]*

[1] Department of Physiology, Semmelweis University School of Medicine, Budapest, Hungary
* Correspondence: jakus.zoltan@semmelweis.hu; Tel.: +36-1-4591500 x60429

Supplementary Material

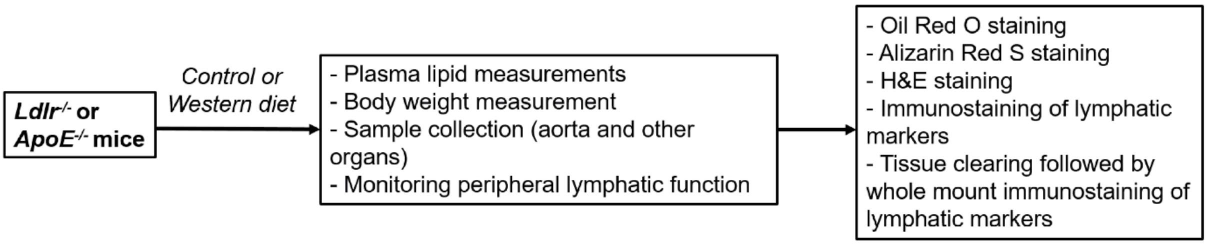

Supplementary Figure S1. Experimental setup. *Ldlr*$^{-/-}$ and *ApoE*$^{-/-}$ mice were fed a control or Western diet for 20-30 weeks after which plasma lipids, body weight and peripheral lymphatic function were measured. Aorta of both *Ldlr*$^{-/-}$ and *ApoE*$^{-/-}$ mice on control and Western diet were collected, followed by Oil Red O staining, Alizarin Red S staining, H&E staining, immunostaining of lymphatic markers, tissue clearing and whole mount immunostaining of lymphatic markers.

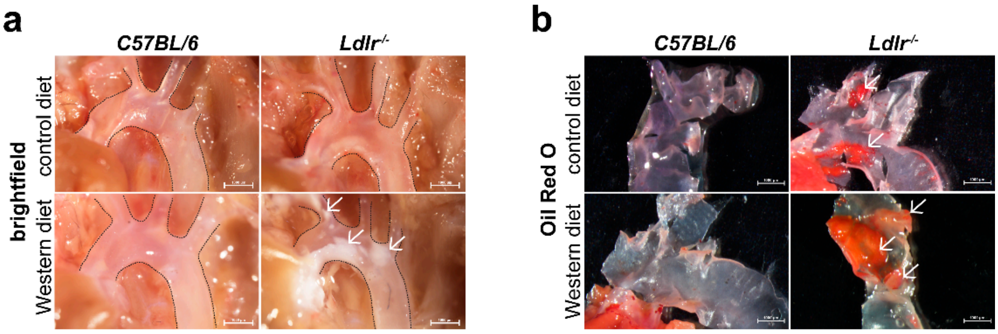

Supplementary Figure S2. Comparison of plaque development in *C57BL/6* wild-type and *Ldlr*$^{-/-}$ mice. **a)** Brightfield images of the aortic arch of female *C57BL/6* wild-type and *Ldlr*$^{-/-}$ mice in situ after 20 weeks on control or Western diet. Images were acquired by stereo microscopy, scale bar = 1000 µm; n = 3 aortas of 3 mice per group. Arrows indicate plaque formation in the aortic arch. **b)** Oil Red O staining of whole aortas of female *C57BL/6* and *Ldlr*$^{-/-}$ mice after 20 weeks on control or western diet. Arrows indicate lipid deposition in the aortic arch. Images were acquired by stereo microscopy, scale bar = 1000 µm; n = 3 aortas of 3 mice per group.
